# Deep learning based fetal distress detection from time frequency representation of cardiotocogram signal using Morse wavelet: research study

**DOI:** 10.1186/s12911-022-02068-1

**Published:** 2022-12-14

**Authors:** Yared Daniel Daydulo, Bheema Lingaiah Thamineni, Hanumesh Kumar Dasari, Genet Tadese Aboye

**Affiliations:** 1 Department of Biomedical Engineering, Dilla University Referral Hospital, Dilla, Ethiopia; 2 School of Biomedical Engineering, Jimma Institute of Technology, Jimma University, Jimma, Ethiopia; 3 Department of Electronics and Communication, Rayalaseema University, Kurnool, Andhra Pradesh India

**Keywords:** CTG, FHR, Fetal distress, Morse wavelet, Deep learning, Resnet50

## Abstract

**Background:**

Clinically cardiotocography is a technique which is used to monitor and evaluate the level of fetal distress. Even though, CTG is the most widely used device to monitor determine the fetus health, existence of high false positive result from the visual interpretation has a significant contribution to unnecessary surgical delivery or delayed intervention.

**Objective:**

In the current study an innovative computer aided fetal distress diagnosing model is developed by using time frequency representation of FHR signal using generalized Morse wavelet and the concept of transfer learning of pre-trained ResNet 50 deep neural network model.

**Method:**

From the CTG data that is obtained from the only open access CTU-UHB data base only FHR signal is extracted and preprocessed to remove noises and spikes. After preprocessing the time frequency information of FHR signal is extracted by using generalized Morse wavelet and fed to a pre-trained ResNet 50 model which is fine tuned and configured according to the dataset.

**Main outcome measures:**

Sensitivity (Se), specificity (Sp) and accuracy (Acc) of the model adopted from binary confusion matrix is used as outcome measures.

**Result:**

After successfully training the model, a comprehensive experimentation of testing is conducted for FHR data for which a recording is made during early stage of labor and last stage of labor. Thus, a promising classification result which is accuracy of 98.7%, sensitivity of 97.0% and specificity 100% are achieved for FHR signal of 1st stage of labor. For FHR recorded in last stage of labor, accuracy of 96.1%, sensitivity of 94.1% and specificity 97.7% are achieved.

**Conclusion:**

The developed model can be used as a decision-making aid system for obstetrician and gynecologist.

## Background

Fetal distress is a condition that results insufficiency of oxygen reaching to tissue and rise of the fetus body fluid acidity condition [1]. If the situation is not intervened immediately it can cause a series damage to brain of the fetus [2] or prenatal death [3].Cardiotocography is the most common noninvasive device used to monitor and evaluate the condition of fetus during labor and pregnancy. Clinically CTG signal is interpreted by experts visually using guidelines [4].

Despite the fact that clinical guidelines are available for interpretation of CTG, there exist a significant inter-observer variability among experts in visual interpretation [5, 6]. In order to interpret CTG accurately experts need to have experiences, unless very crucial patterns may be missed or interpreted wrongly. Misinterpretation of CTG signal may lead to unnecessary surgical delivery [7].

To overcome the problems in visual interpretation several studies have done to develop computer aided diagnosing models using varies method. Among them rule based system is the one which mainly focus on identifying morphological parameters defined in the clinical guidelines [8–10]. The major shortcoming associated with the most rule based systems is that they focus on identifying the morphological features rather than classifying the signal in to normal and abnormal. Moreover, the rule-based system necessitates extensive domain knowledge as well as a significant amount of manual work, making it time-consuming.

Development of automated model for CTG signal analysis extended from identifying the guideline features to approach of extracting of varies domain feature and selecting the most important features for classification [11–13]. And this method of automated classification is called conventional machine learning approach. Comert et al. [14] used time–frequency image of FHR signal to develop novel prognostic model using machine learning for assessment of fetal distress. Another study was conducted by Zafer et al. [15] on evaluation of fetal distress diagnosis for FHR recorded in first and second stage of labor using conventional machine learning approach.

Generally, in conventional machine learning approach, hand crafted feature extraction strategy applied to extract the most important features that conveys information about fetal distress is still challenging. Typically, more features even that are irrelevant to the task at hand may be extracted or small features may be selected and this lead to loss of valuable features. To overcome this problem CAD based CTG signal classification are inspired by art of deep learning which differs from traditional machine learning in a way that it does away with the need for handcrafted features by learning valuable features directly from data, eliminating the need for manual feature extraction and selection approaches. Most of deep learning model requires 2D image data as an input however, the raw CTG signal is 1D time series signal and various techniques were applied in related works to convert the 1D CTG signal to 2D.

Bursa et al. [16], developed convolutional neural network model which aimed to classify CTG signal. They used a continuous wavelet transform family named complex Morlet for generation of time frequency image of FHR signal to feed the CNN model. The model is tested on FHR data that is recorded during stage I labor and a classification accuracy of 94.1% were achieved by this model. A transfer learning strategy, a deep convolutional neural network was developed using AlexNet model by Comert et al. [17]. They used STFT to generate time–frequency image of FHR signal to feed the AlexNet model and achieved classification accuracy of 94.32%. Another deep learning model was developed by Zhao et al. [18]. They used CWT family named db and sym to generate input images for the deep learning model. An optimized ResNet 50 convolutional neural network was implemented for assessment of maternal and embryo risk during pregnancy [19]. They used various time domain features extracted using conventional machine learning approach to feed the deep learning model. After extraction of feature optimized ResNet was used for classification and the achieved accuracy of 94.63%.

As stated on the aforementioned related work, time–frequency representation of FHR signal using varies technique were implemented to generate an input image for deep learning model and promising classification accuracy were achieved. In some literatures [20–22], scanned image of CTG signal were also used to generate 2D image as input for deep learning models. Detection of preventable fetal distress using deep learning approach from scanned image of CTG signal was performed in [20]. A private data was used in their study and they achieved classification accuracy of 93.65%. Deep learning model named CTG-net was developed in [21] for classification of scanned image CTG signal and they reported area under the receiver operating characteristic curve of 0.73**.** Another study was conducted on fetal distress classification with deep convolutional network by Saini et al. [22]. They used scanned image of CTG signal to classify fetal distress in to three class named as normal, mild and severe.

Most of related work which were conducted based on deep learning approach shown very good potential in classification of fetal distress, however the accuracy achieved in [21] and [22] was very low. Even though highest accuracy was achieved in [17] and [18] application of the models and time–frequency representation of FHR for various stage of labor were not conducted and is questionable. Moreover, in the study of [20], pre-classification of the signal was done visually which is highly susceptible to high false positive. In [16] the model has not yet been fully validated and there is a problem on performance analysis of the models.

In this study, gaps of aforementioned works were addressed through taking positive motivational methodologies and considering the critical factors that affects accurate detection of fetal distress from FHR signal of CTG.

The key contribution of the current study is as follows; preprocessing done in the first step on the raw FHR signal was to remove unwanted artifacts and missing data. Converting the 1D FHR signal to 2D image by applying effective method of time–frequency representation was done in the second step. Fine tuning of the ResNet 50 deep learning model for training and classification was done in the third step. The last step was used to conduct comprehensive experiment on FHR signal that were recorded on early (first stage) and last (second) stage of labor. Since the quality and nature of FHR signal varies depending on the labor stage when it was recorded [23], a comprehensive experiment has been done for FHR signal recorded in varies stage of labor to confirm robustness of the model.

## Methods

### Data source

The CTU-UHB data which were obtained from the Physio Net is the only and the largest open access database of its type. The data base consists of 552 recording which were obtained in the university's obstetrics ward in Brno, Czech Republic. All recordings are 90 min in length and begin 90 min prior to delivery. The database consists of simultaneous record of UC and FHR which are sampled at 4 Hz. The CTG signal recording lasts 60 min for 1st stage of labor and 30 min for 2nd stage [24].

**Fig. 1:**
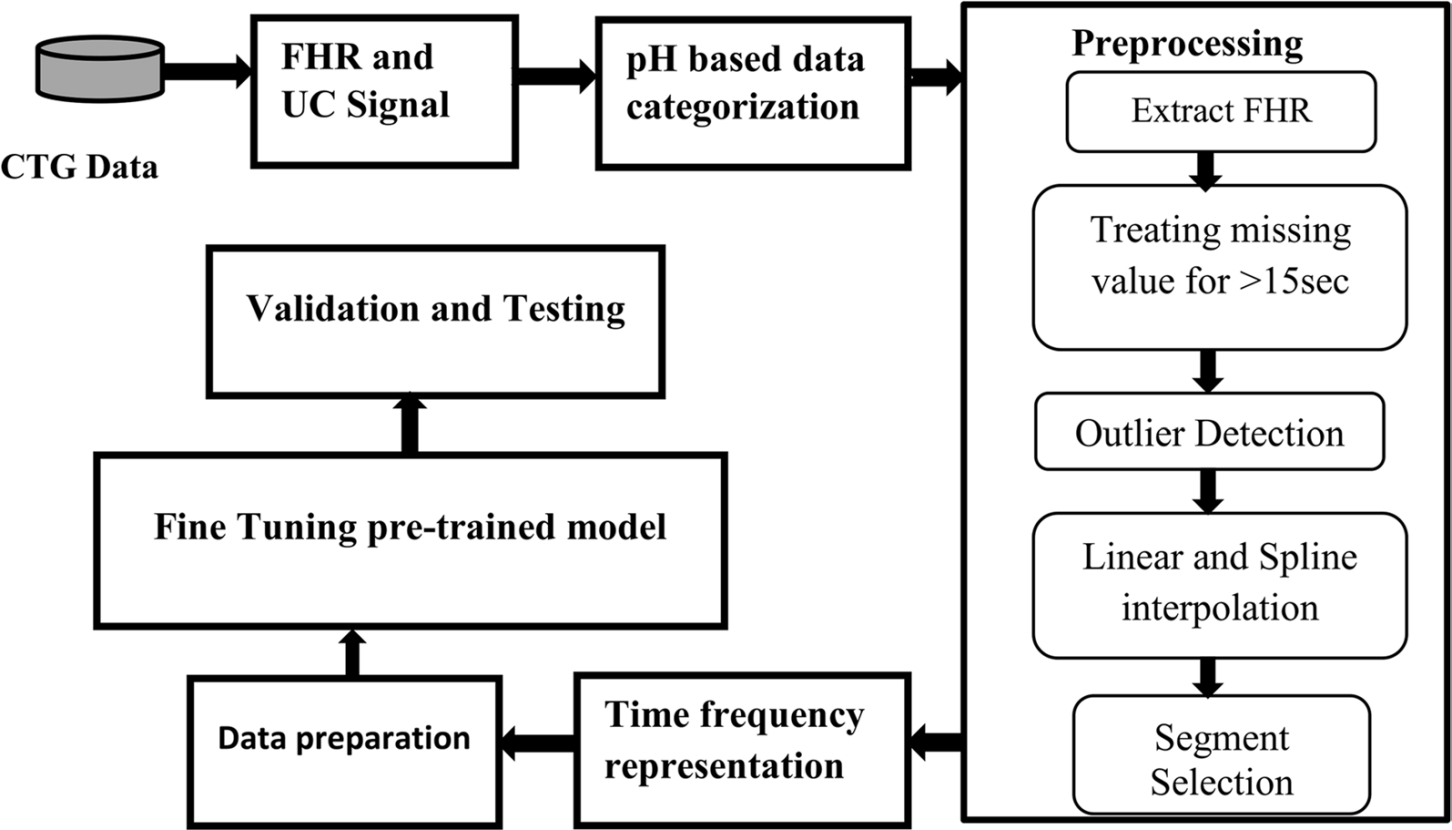
Schemes of overall methodology

Figure 1 show the general schemes of the methodology followed in this study and it includes: labeling of the CTG signal, preprocessing, time frequency representation, data preparation for training of mode, fine tuning a pertained Resnet50 model and evaluation of the model performance through validation and testing. There are several methods of data pre classification or data labeling criteria for CTG signal analysis. Among them, labeling based on experts visual annotation, APGAR score [8] and pH based annotation [15–18] are the most common methods. Since clinical expert’s visual annotation and APGAR score are subjective data labeling criteria, so both were disregarded and the objective data labeling criteria that is pH value of neonatal umbilical artery blood measured shortly after the baby was born [25] is used. So, a pH less or equal to 7.15 was decided to be pathological and a pH greater than 7.15 is assigned for normal class after careful examination. Based on our data pre classification criteria, the database contained 439 normal and 113 for distressed classes.

### Preprocessing

In biomedical signal processing and analysis, preprocessing is the first and very important step to be made before further analysis of the signal. Nature of features used in training and final classification of the model is reliant on the signal quality obtained after preprocessing [14].

**Fig. 2:**
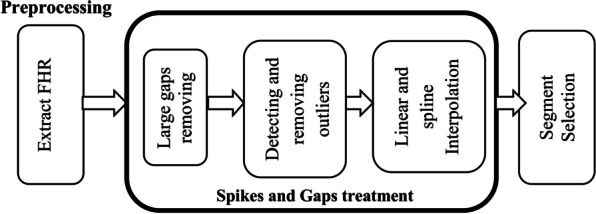
Steps of preprocessing

In clinical practice CTG signal is acquired by external sensors, thus FHR signal is subjected to artifacts and spikes that arise from maternal movement, sensor displacement which may cause the signal drop to zero value [11] and other deliver related factors [18]. The noises that affects FHR signal ordinarily reveals itself as spiky, outliers or missing value (periods where FHR value drop to zero). Before further analysis of the CTG signal, noises are eliminated to obtain reasonably better quality signal for more accurate results. So, the main goal of this step is to reduce the aforementioned noises by applying a conventional preprocessing techniques applied in FHR signal analysis [13, 26] and the steps are shown in Fig. 2.

First the raw CTG signal containing FHR and UC signal are obtained, and then FHR signal is extracted and passed to the further step. Then the long gaps which is a missing value for more than 15 s are removed from the time series signal [20]. In addition, missing values at the beginning and at the end of recording are excluded to start from the stable point. The extreme value of FHR that are less than 50 bpm and greater than 200 bpm are called outliers (not physiologic) [17]. The outliers and small gaps are determined and linearly interpolated [13] using an algorithm provided by Matlab 2021a. It is a type of interpolation which uses linear polynomial to generate a new data point between two points by using curve fitting technique. For a two known data points by coordinates of (x_0,_ y_0_) and (x_1,_ y1) linear interpolation generates the new value of **y** using Eq. 1.

1 $$y = \frac{{y_{{0{ }}} \left( {x_{1} - x} \right) + y_{{1{ }}} \left( {x - x_{0} } \right)}}{{x_{1} - x_{0} }}$$

**Fig. 3:**
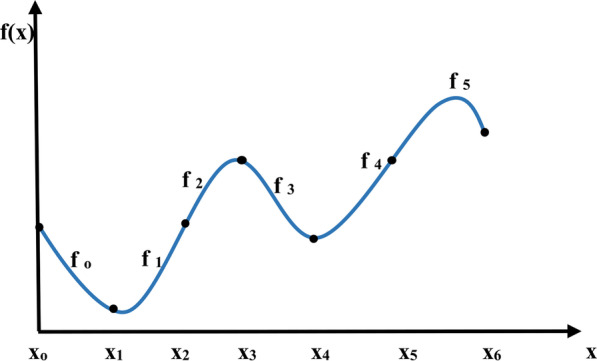
Cubic spline interpolation

Sample points of FHR signal that is greater than by 25 beat from the previous adjacent beat is not physiologic and unreliable beat [27] and this unstable point reveals itself as spikes on a FHR signal, so it is removed using cubic spline interpolation [13]. It is a very powerful and widely used method that interpolates a function between a given set of points by means of piecewise smooth polynomials [28]. A cubic spline f(x) interpolating on the partition x_0_ < x_1_ < ⋯ < x_n-1_ is a function for which f(x_k_) = y_k_ is a piecewise polynomial function that consists of n − 1 cubic polynomials f_k_ defined on the ranges [x_k,_ x_k+1_]. An example of cubic spline interpolation passing through 6 data point is shown in Fig. 3.

**Fig. 4:**
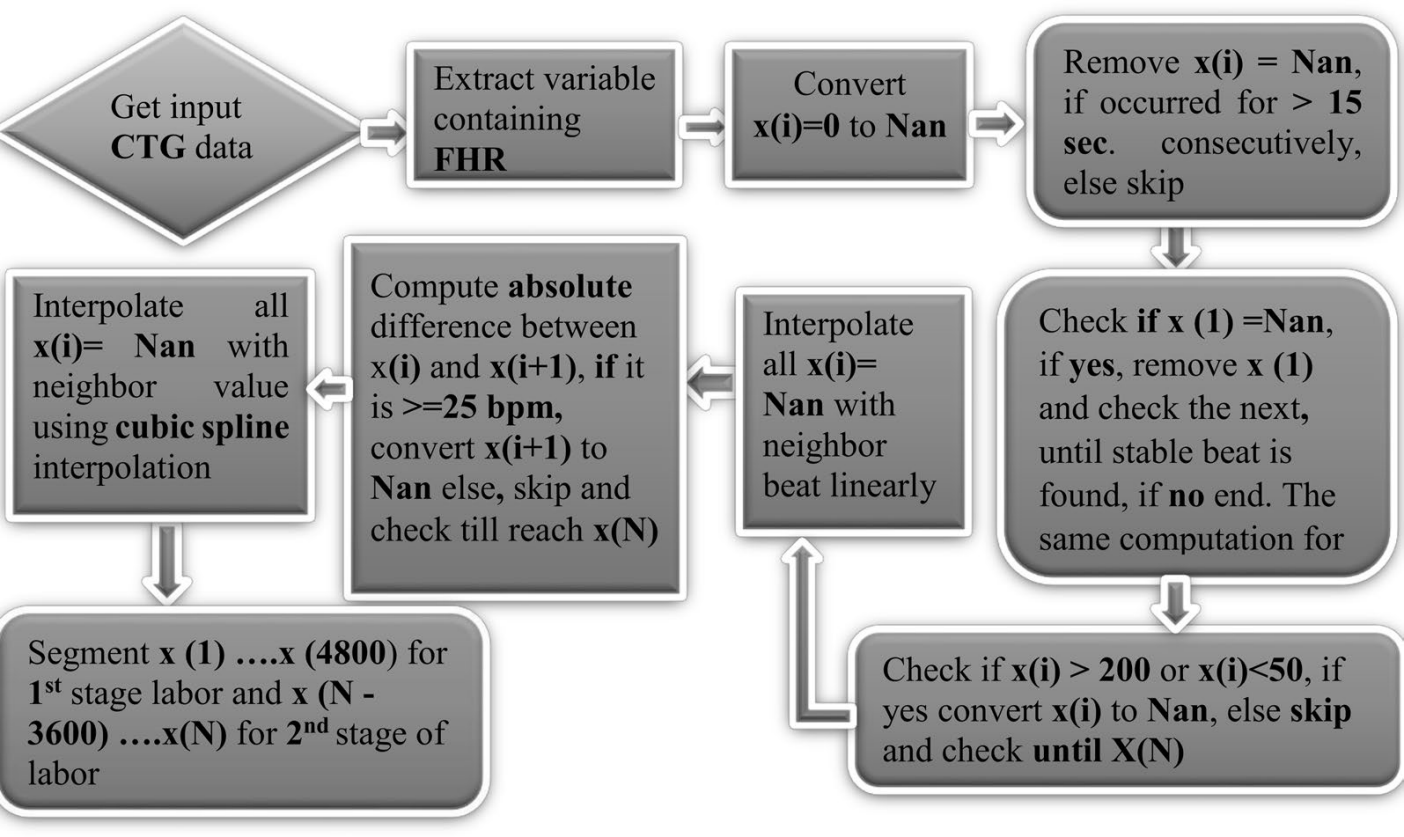
Algorithm applied in preprocessing of FHR signal

Finally segment of the 1st 20 min (4800 sample) [12, 13, 18] and last 15 min (3600 sample) [17, 29] were selected for further analysis considering the length of signal in first and second stage of labor. Considering x(*i*) as FHR signal having a sampling frequency of 4 Hz and a unit of beats per minute (bpm), where *i* = 1,2, …, *N* and *N* is the number of sample points, the following logic shown in Fig. 4 is performed in preprocessing stage using Matlab 2021 a.

**Fig. 5:**
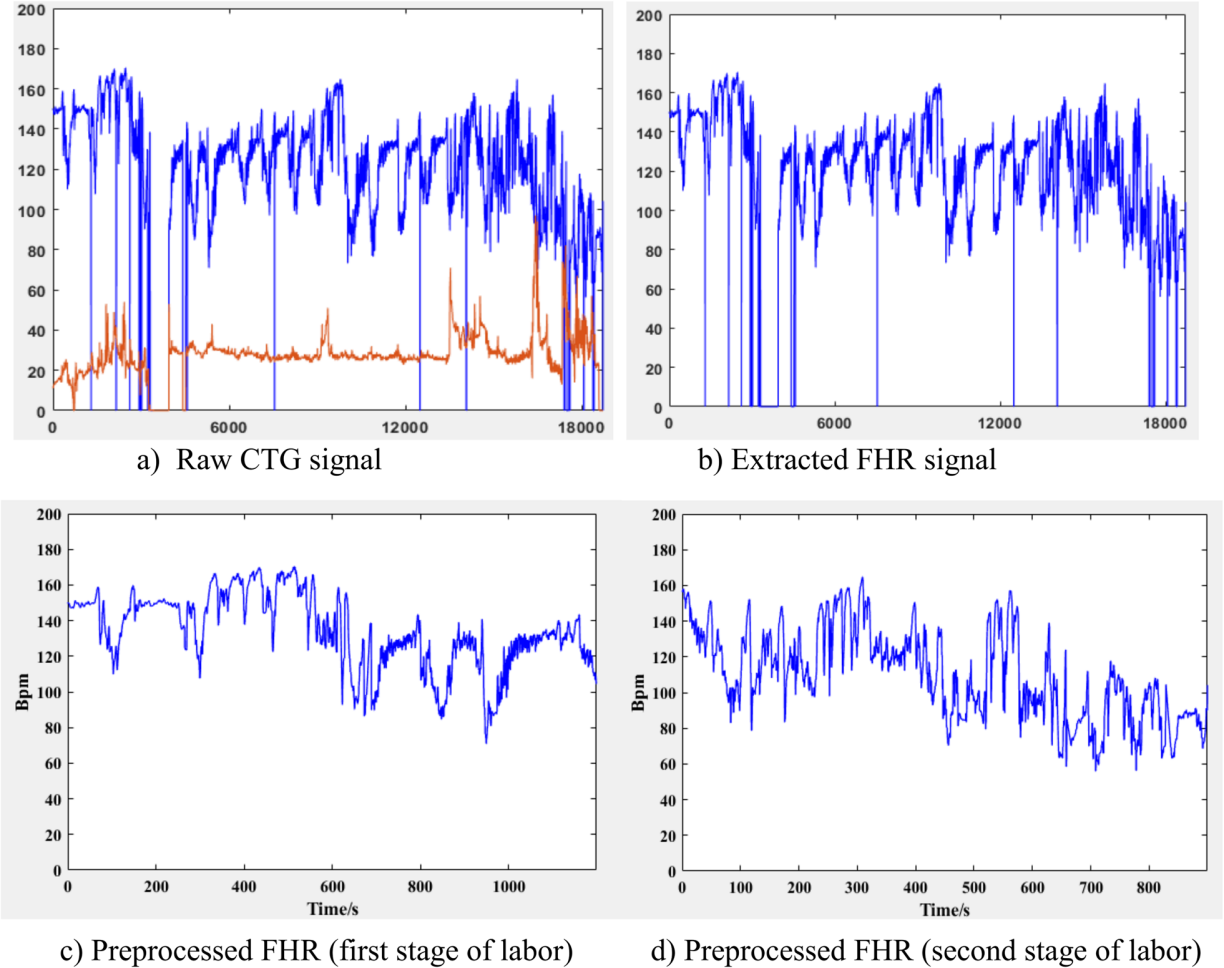
Preprocessing stages of CTG signal

In stage of preprocessing, first the FHR signal is extracted as shown in Fig. 5b from the CTG signal that contains the FHR and uterine contraction signal as described Fig. 5a. Once the FHR signal is extracted it goes to further stage of preprocessing and finally segmented based on stage of labor as early stage of labor and final stage of labor for experiment one and two as shown in Fig. 5c and d respectively.

### Data augmentation

One of the most frequent problems in the field of deep learning is unbalanced classes. Data augmentation is one of the ways for dealing with this problem. Bearing in mind the amount of data available, under-sampling the largest class (Normal class) was ruled out and over-sampling the minority distressed class was chosen for augmentation. There are some types of time series data augmentation inspired from 2D augmentation such as: Jittering(injecting noise), rotation, scaling, window slicing [30]. Slicing augmentation is implemented in this work; it is the same as data augmentation used for image called cropping augmentation. The main idea underlying slicing is that the data is augmented by removing or adding time steps from the pattern's ends. Likewise, minority class of database is oversampled in the time series by slicing shifting backward for five minute two times. Therefore, slice of 20-min window slice for first stage and 15-min window slice for second labor stage data were generated.

### Time frequency representation of FHR signal using Morse wavelet

Various types of wavelet family are available for CWT, among them analytic wavelet (AW) has been widely employed for time frequency analysis and representation of physiological signal such as electroencephalogram (EEG) [31], electrocardiogram (ECG) [32], electromyogram (EMG) [33]. It's a wavelet with a complex value and a Fourier transform of zero at negative frequencies [34] and a generalized Morse wavelet is a latest and well known of its family. This family of wavelet is an ideal choice for analysis of non-stationary signal with varying amplitude and frequencies over time. It calculates the amplitude, frequency, transient, short duration, localizing discontinuities, and combined time–frequency localization of time-varying amplitude, frequency, transient, and short duration [35].

From the family of analytic wavelet specifically generalized Morse wavelet is called exactly analytic wavelet as it has no leakage for negative frequency unlike other analytic wavelets [36]. Negative frequency leakage in wavelets causes interference and degrades the transform result [33, 35]. Furthermore the flexibility nature of generalized Morse parameters made it a super family to encompasses all other analytic wavelet class [36, 37].

Generalized Morse wavelet form has two parameter exhibiting additional degree of freedom in comparison with other AW. It is represented as $\varphi_{P,\gamma } \left( t \right)$ and defined in frequency domain [36, 38] by Eq. 2

2 $$\varphi_{P.\gamma } \left( \omega \right) = U\left( \omega \right)\alpha_{P,\gamma } \omega^{{\frac{{P^{2} }}{\gamma }}} e^{{ - \omega^{\gamma } }}$$

P^2^ is time bandwidth product, $\gamma$ is symmetry parameter, is Euler’s number $\approx 2.71828$, $U\left( \omega \right)$ is unit step function and $\alpha_{P,\gamma }$ is normalizing constant. Rather than the time bandwidth product, $\beta$ is employed as a decay or compactness parameter in several Morse wavelet applications which is $P^{2} = { }\gamma { *} \beta$ [35]. The equation of Morse wavelet using $\beta and \gamma$ is written as Eq. (3).

3 $$\varphi_{\beta .\gamma } \left( \omega \right) = U\left( \omega \right)\alpha_{\beta ,\gamma } \omega^{\beta } e^{{ - \omega^{\gamma } }}$$

By adjusting $\gamma$ and $\beta$ parameters, the generalized Morse wavelet can take a broad range of mother wavelet that has not been even fully explored [35]. For instance setting $\gamma$ = 1 and $\gamma$ = 2 results to other family of analytic wavelet named Cauchy and derivative of Gaussian wavelets respectively [36].

The wavelet duration in time is proportional to the square root of the time bandwidth product P and determines number oscillation that can fit into the envelop [37], whereas the symmetry parameter $\gamma$ determines symmetry behavior of wavelet in time domain [33].

When ${\upgamma }$ is set to 3, the skewness of the Morse wavelet via demodulation is 0 and this value results the wavelet to exhibits minimum Heisenberg area while remaining exactly analytic [35]. Wavelet with large Heisenberg area results to poor time frequency concentration [37], so setting $\gamma$ = 3 results the wavelet to the most symmetric and the most Gaussian wavelets (‘Airy’ wavelet family) with minimum of Heisenberg area [33]. Hence the value of symmetry parameter $\gamma$ = 3 is used for time frequency representation of FHR in this work.

**Fig. 6:**
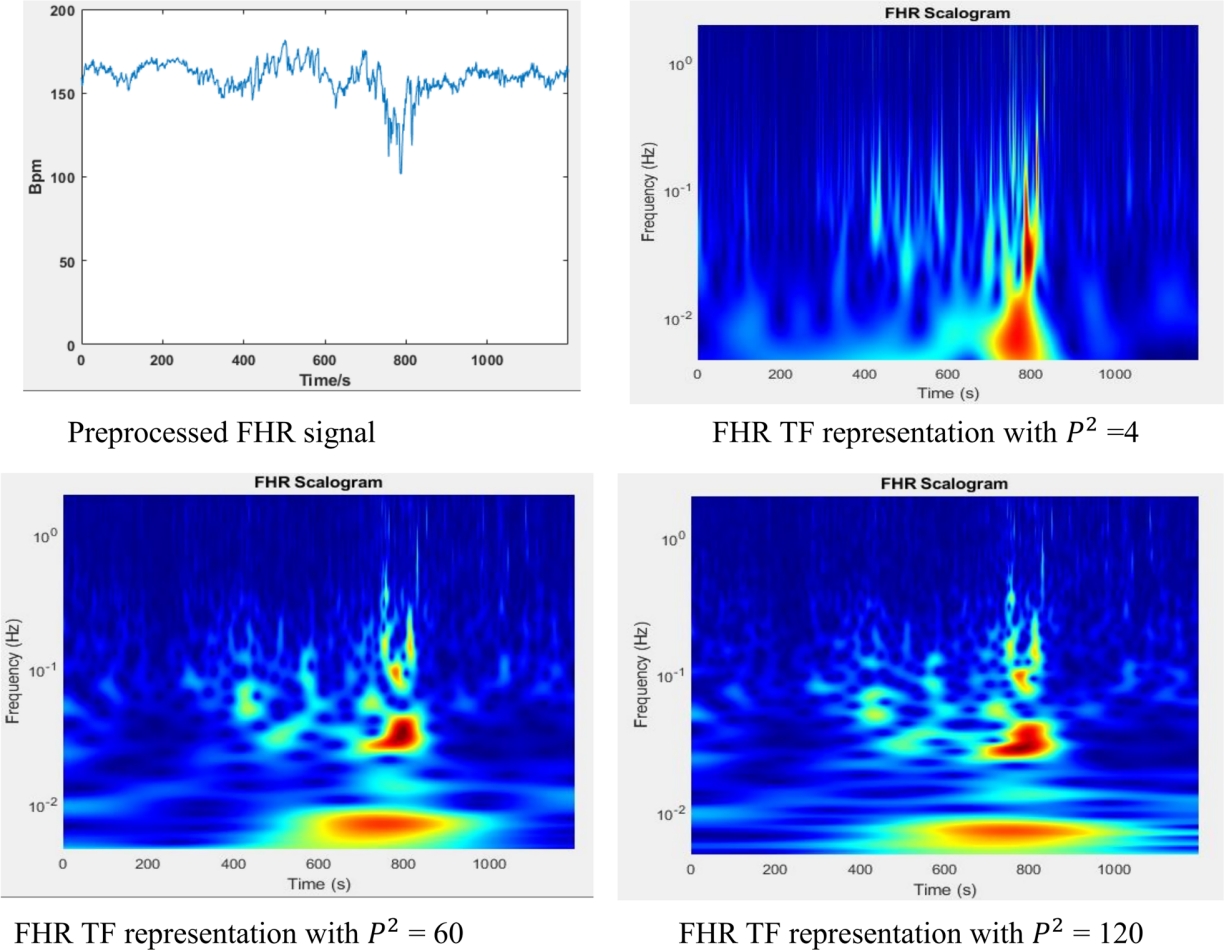
Effect of generalized Morse parameter on time frequency trade off

As discussed previously time-bandwidth product determines oscillations in the envelope [38]. so, for a fixed value of $\gamma$ at 3 varying time-bandwidth product $P^{2}$ varies the oscillatory behavior of wavelet. Therefore, based on type of analysis and behavior of signal, one can adjust Morse parameters value and examine its effect on the mother wavelet and on frequency response of filter bank. Figure 6 shows the effect of time different value of time-bandwidth product value for fixed value of $\gamma$ at 3.

As shown in Fig. 6 when the value of time-bandwidth product $P^{2}$ increases the wavelet becomes more oscillatory in the envelop, this leads the wavelet to become narrower in frequency and spread out in time. The plot of frequency response clearly visualizes the effects in frequency domain, for the value of $P^{2}$ = 4 the frequency response is widest compared with $P^{2}$ = 60 and 120. At $P^{2}$ = 120 it’s very narrow.

The value of Morse wavelet parameter determines time frequency tradeoff in representing the FHR signal. For lower $P^{2}$ value the wavelet transform results in good temporal resolution but poor spatial resolution, whereas for higher $P^{2 }$ the wavelet transform becomes good in spatial resolution but poor resolution in temporal. For time frequency analysis the default value of time-bandwidth product which is 60 is recommended [32]. Considering the effect in time frequency trade off, the extreme values of $P^{2}$ is ruled out for this study, as the aim is to localize visible and hidden characteristic of FHR in time and frequency jointly. So, time band width product value of 55 and 60 with symmetry parameter $\gamma$ = 3, are carefully selected for time frequency representation of FHR signal in this work. A Matlab ‘cwtfilterbank’ is utilized for time frequency conversion of FHR signal. The parameters in the filter bank are adjusted considering the FHR signals and wavelet parameters, thus sampling frequency 4 Hz, signal length (4800 and 3600) gamma parameter 3 and time bandwidth product value 60, 55 and voice for octave value of 12 are used.

### Adoption of pre-trained ResNet 50 model for transfer learning

Transfer learning is a term that refers to passing weight values of a trained neural network to another new neural network, so that building and training a network from scratch will be avoided [39]. Transfer learning makes updating and retraining a network considerably faster and easier than training a network from scratch. It permits the use of popular models that have already been trained on huge datasets to train models with less labeled data and some of the well-known CNN models used for transfer learning are; AlexNet, GoogLeNet, Vgg, OverFeat, ResNet, Xception [40]. Different aspects of pre-trained networks are important to consider when selecting a network to use for a specific purpose. Network accuracy, speed, and size are the most critical aspects. However, choosing a network is usually a compromise between these factors. So, taking major consideration such as limited infrastructures like memory space and GPU and accuracy of the model in account ResNet 50 is selected for this study.

**Fig. 7:**
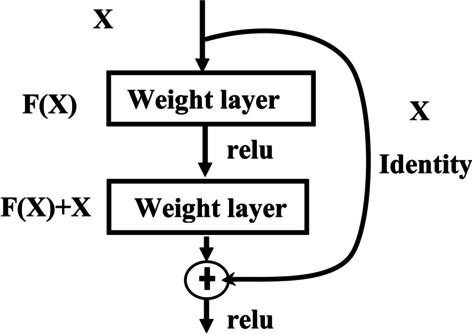
Residual learning building block

ResNet is a sort of neural network first introduced by K He et al. in 2015 [41]. It is an architecture designed to be more in-depth structured than all previous architectures and enables for the successful training of incredibly deep neural networks without being hampered by vanishing gradients [41]. ResNet counters the problem of vanishing gradients by introducing identity shortcut connection indicated by X in Fig. 7 or skip connection indicated curved arrow in Fig. 7.The identity connections helps the residual block to reuses the input features of the upper layer and add it with output of current layer before feeding the next layer as illustrated in Fig. 7.

**Fig. 8:**
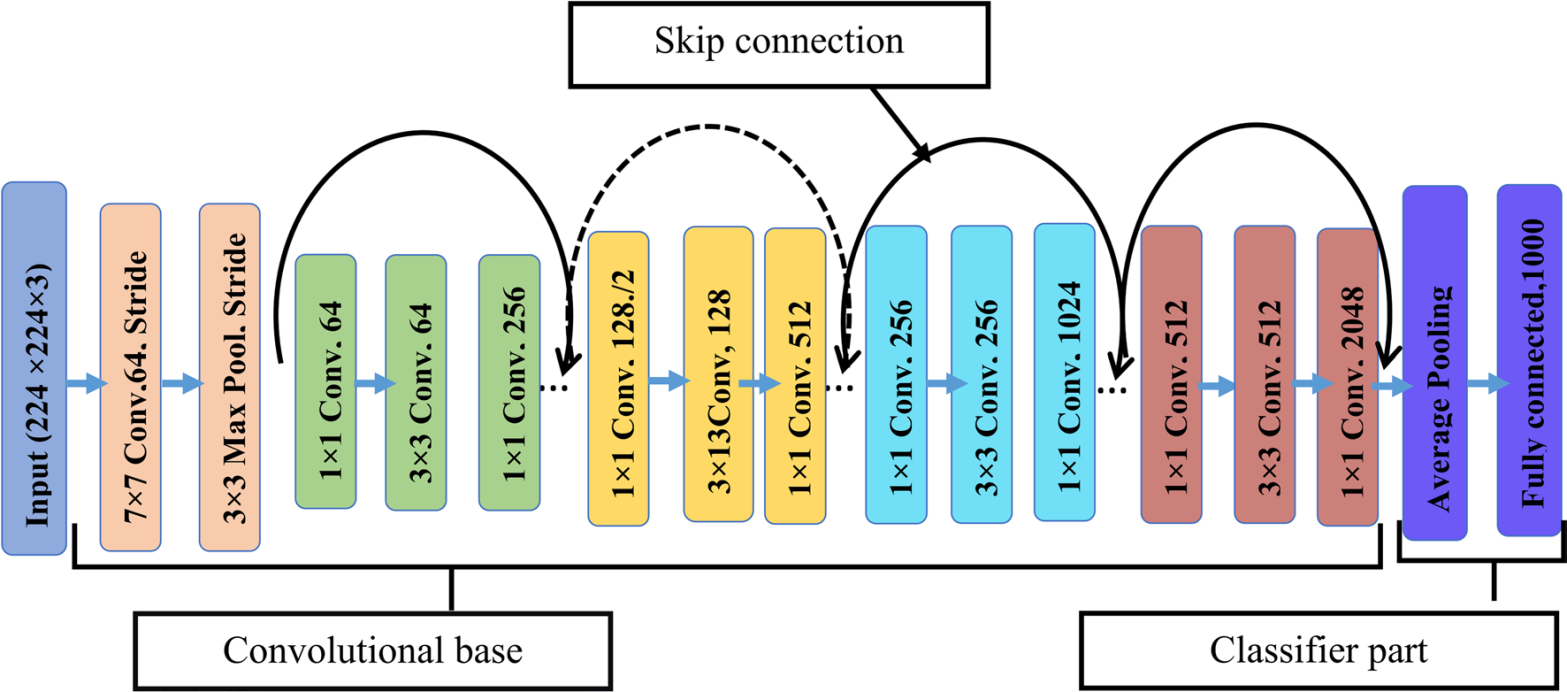
Architecture of ResNet 50

ResNet is one of the most powerful deep neural networks, which achieved exceptional generalization capabilities on ILSVRC 2015 classification competition. ResNet also took first place in the ILSVRC and COCO 2015 contests for ImageNet detection, ImageNet localization, COCO detection, and COCO segmentation [42]. ResNet50's design is divided into four stages, as shown in Fig. 8 the network uses 7 × 7 and 3 × 3 kernel sizes for initial convolution and max-pooling respectively on input image of size 224 × 224 × 3. Following that, Stage 1 of the network begins, which consists of three residual blocks, each block has three layers, and kernel sizes utilized to execute the convolution operation in all three layers of the stage 1 block are 64, 64, and 128 [42].

### Data split scheme

The dataset split was done for training and testing by applying a well-known rule of data splitting which is 80% and 20% training and testing sets respectively. Among the 20%, 10% was used for validation, and 10% for test. A training set is used to train the network while a validation set is used to monitor the model performance and fine tune hyper-parameters during the training process. Finally, a test set is used once in order to evaluate the performance of the final model.

## Result

### Training result

After augmentation final dataset contained 1556 time frequency images of which 878 is for normal class and 678 for pathologic or distressed class. As per the data split ratio used, the number of data used for training the system is 790 for normal and 610 for distressed. The remaining 10% which is 156 of which 68 for pathological and 88 for normal were taken as testing set. During training 10% of training data from each class was randomly selected based on validation frequency set which is 15 iterations. Once data split and preparation were complete the ResNet50 model is trained separately for first and second experiments which are the first 20 min and the last first 15 min of the CTG recording. The ResNet-50 model performance is evaluated to select the best fine tuning parameters to do the classification task. Hence, the model was trained using the training dataset and validated with the validation dataset.

**Fig. 9:**
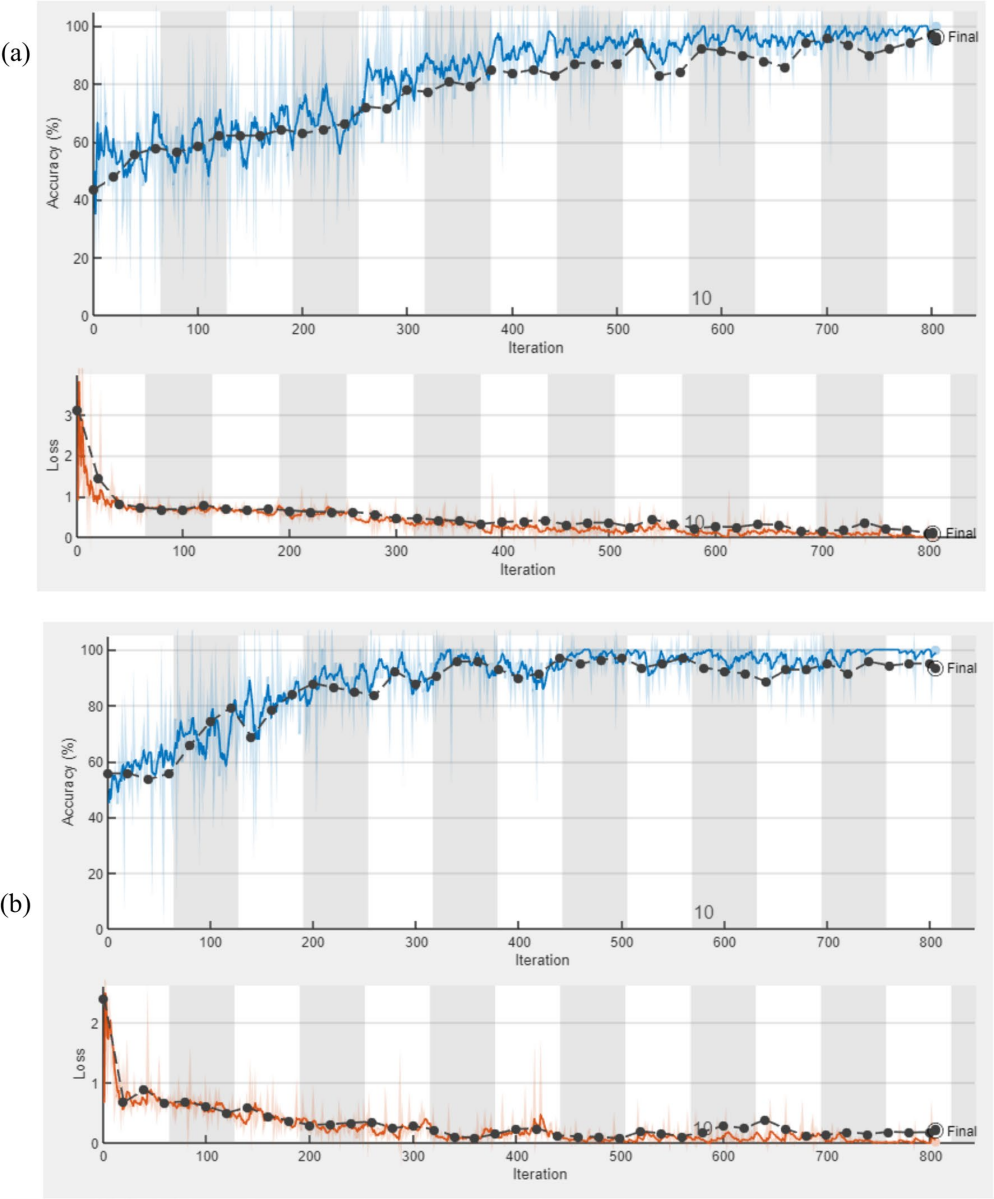
Training and validation curve of ResNet for 1st stage (**a**) and 2nd stage (**b**)

The learning curve is derived from the training dataset and depicts the model's learning ability. The validation learning curve, on the other hand, is derived using a validation dataset to determine how effectively the model generalizes. Figure 9 demonstrates the curve plots of the training accuracy (blue curve), validation accuracy (black dot with blue curve), training loss (brown curve) and validation loss (black dot with brown curve) of models.

**Fig. 10:**
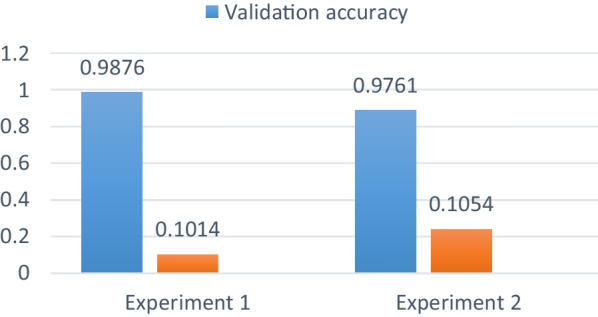
summery of training performance

During training, the lowest validation loss achieved were 0.1014 for Experiment 1 and 0.1054 for experiment 2. And the validation accuracy of 98.76% and 97.61% were achieved for experiment 1 and experiment 2 respectively as summarized in Fig. 10.

### Testing result

The performance of the model was evaluated for different performance metrics such as classification accuracy, sensitivity and specificity. In classification the model is expected to categorize given input data into normal and distressed class. In order to do this 10% from each class that was already preserved for testing is used, among the testing set 68 data’s were assigned for distressed and 88 data’s for normal classes.

According to the confusion matrix for experiment 1 in Table 1a two of the ‘Distressed’ class are misclassified as ‘Normal’ class and all ‘Normal’ class are classified correctly. For second experiment in Table 1b, four of the distressed classes are misclassified as normal and two of the normal classes are misclassified as distressed class. From the confusion matrix result in Table 1 TP, TN, FP and FN values are easily known and the performance evaluation metrics are calculated, thus accuracy of 98.7%, sensitivity of 97.0% and specificity 100% are achieved for FHR signal of 1st stage of labor and accuracy of 96.1%, sensitivity of 94.1% and specificity 97.7% are achieved for FHR signal of 2nd last stage of recording.

## Discussion

Among the noninvasive devices used to monitor fetal heart activity, cardiotocography is the widely used technique for monitoring FHR and diagnose fetal distress. It is one of the most frequent ways for assessing fetal well-being during pregnancy and birth, and it aids in the detection of potential fetal risks such as hypoxia and distress. According to assessment conducted on inter observer agreement, visual interpretation of CTG signal suffers from observer variability that results to low inter-observer agreements. The variability in visual interpretation, emphasizes that automating fetal distress diagnosis is very important for reducing diagnosis errors that are brought through the current traditional manual diagnosis technique. To achieve this FHR signal is preprocessed and converted to time–frequency representation using generalized Morse wavelet. Then a pre-trained ResNet 50 model was fine-tuned on FHR data of first 20 min and the last 15 min.

In preprocessing large gaps which are missing for 15 s were completely removed from the signal. Cubic spline and linear interpolation was used in preprocessing to remove noises, outliers and missing values and unreliable beats. After preprocessing, the FHR signals were represented using generalized Morse wavelet with gamma parameter γ = 3, which is a value for the most symmetric and the most Gaussian wavelets (‘Airy’ wavelet family) [40], with minimum of Heisenberg area. In addition, the effect of time bandwidth product P parameter on frequency response of filter bank and on FHR time frequency representation were examined for γ value fixed at 4. As a result of raising the time bandwidth product P, the wavelet filter bank frequency response was narrowed in frequency while the breadth of the middle component of the filter was increased in time as described in Fig. 6, due to these effects on filter bank the higher *P* value results to high frequency resolution with poor time resolution, whereas lowering *P* value results to high time resolution with poor frequency resolution. Thus considering the frequency range of FHR and effects on time frequency representation, *P* value of 55 and 60 was used to generate an input time frequency image for the pre trained Models.

After fine tuning the ResNet50 model its performance is examined during training. During training the performance evaluation of the model is done using learning curve by examining two different metrics which are the accuracy and loss metrics. For both training and validation datasets, a good result is attained when the accuracy curve grows and the loss curve lowers as the number of epochs increases. As a result, both metrics plots were created for each experiment and by reviewing the learning curves shown in Fig. 9a and b the performance of the model was evaluated.

When examining the performance for both experiments, The ResNet 50 model fine-tuned with Adam optimizer, learning rate 0.001, validation frequency 20 iteration and mini batch size 30 achieved higher validation accuracy at 13^th^epoch which is 98.76% and 97.61 for experiment 1 and 2 respectively. Finally, average test accuracy of the model for classification was calculated. Hence, accuracy of 98.7%, sensitivity of 97.0% and specificity 100% are achieved for FHR signal of experiment one. For second experiment an accuracy of 96.1%, sensitivity of 94.1% and specificity 97.7% are achieved.

The testing result achieved with the ResNet 50 model is comparable with the most related literatures [16, 22], studied on the same CTU-UHB data base and experimented on FHR recorded on early and last stage of labor as described in Table 2. Comparing with [16], FHR and UC time frequency information of first labor stage has been used using Complex Morlete with varies detail parameter to increase the dataset. y. In [17], STFT was used for time frequency representation of second stage FHR data and four time–frequency image generated for one FHR signal based on frequency range of FHR and this helped to increase the size of database. They used a pre-trained AlexNet model for classification. The main difference of their study with current study is using STFT for time frequency representation and using pH value less 7.05 for discrimination of FHR data normal and hypoxic. They reported average Acc, Se, and Sp of 93.32%, 56.15%, and 96.51%, respectively. Even though a promising result was achieved for second stage of labor, the model is complex to implement as the time frequency representation is done based on the four different frequency ranges which is associated with a varies physiological events. Moreover, the pH value less 7.05 which is assigned for the pathological class is representative of severe hypoxic fetus, thus the model may classify mild hypoxic fetus in to normal. In [17, 18] CWT two mother wavelet (db and sym) with order of two and scale of 4, 5 and 6 was used for time frequency representation of FHR data of first labor stage and they designed a CNN from scratch on Matlab, thus reported accuracy, sensitivity and specificity of 98.34%, 94.87% and 97.82% respectively. Since a single wavelet scale is used in the study, the time frequency representation fails the multiresolution analysis of CWT and captures limited information within the specified scale and this may lead to low the performance of the model if implemented in second labor stage FHR data.

Moreover, in some literatures the CNN model is fed with scanned image of CTG signal. Accordingly in [19] and [20] classification accuracy of 94.63% and 93.6% were reported respectively. In [21] CTG net CNN model was developed reported AUC of 0.78. In [22] scanned image of CTG signal with a morphological feature was used and accuracy of 70%, 71.4% and 70% for normal, mild hypoxia and severe hypoxia respectively were reported.

Comparing with aforesaid literatures which are implemented on deep learning model with time frequency of representation for FHR such as Complex Morlete [13], STFT [16], db and sym wavelet [17], our model achieved better classification task for first and second labor stage FHR data.

To generalize, desirable behavior of generalized Morse wavelet which are: being exactly analytic wavelet which has no leakage for negative frequency [65], the most Gaussian wavelets (‘Airy’ wavelet family) with minimum of Heisenberg area [33], application for analysis of time varying amplitude, frequency, transient, short duration, localizing discontinuities and joint time–frequency representation [35] and the ResNet 50 architecture key features such as; using batch normalization and bottleneck residual block design to increase the performance of the network, using identity connection to protect the network from vanishing gradient problem [42] supposed to improved performance of our experiment.

## Conclusion

This study proposed an automatic system for fetal distress detection from time frequency information of CTG signal using generalized Morse wavelet. A pre-trained ResNet 50 model was used to classify fetal condition as normal and distressed for first stage and last stage of labor FHR data. The study showed that time frequency representation of FHR signal using generalized Morse wavelet has a significant impact on capturing important features of FHR signal, thus improved the classification performance of identifying fetal distress in various stage of labor. Generally, classification accuracy of 98.7%, sensitivity 97.0% and specificity 100% have been obtained for data of first stage of labor and accuracy of 96.1%, sensitivity 94.1% and specificity 97.7% have been obtained for FHR data of second stage of labor using CTU-UHB dataset. Finally, to ease the use of the system a simple Graphical User Interface is developed. This system can play a significant role in supporting obstetrician and gynecologists during the diagnosis procedures.

In this study class imbalance between normal and distressed class was the main challenge. However, data augmentation technique was implemented on distressed time series FHR signal to deal class imbalance issue. But, to develop more robust and reliable model it’s recommended to collect more data rather than augmenting the minority class. Moreover, the study is limited to classify fetal distress in to normal and distressed, but it is recommended to classify the distressed class to severe and mild class. The aforementioned recommendations can be taken into account for further study which are not investigated and addressed in this work.

**Table 1:**
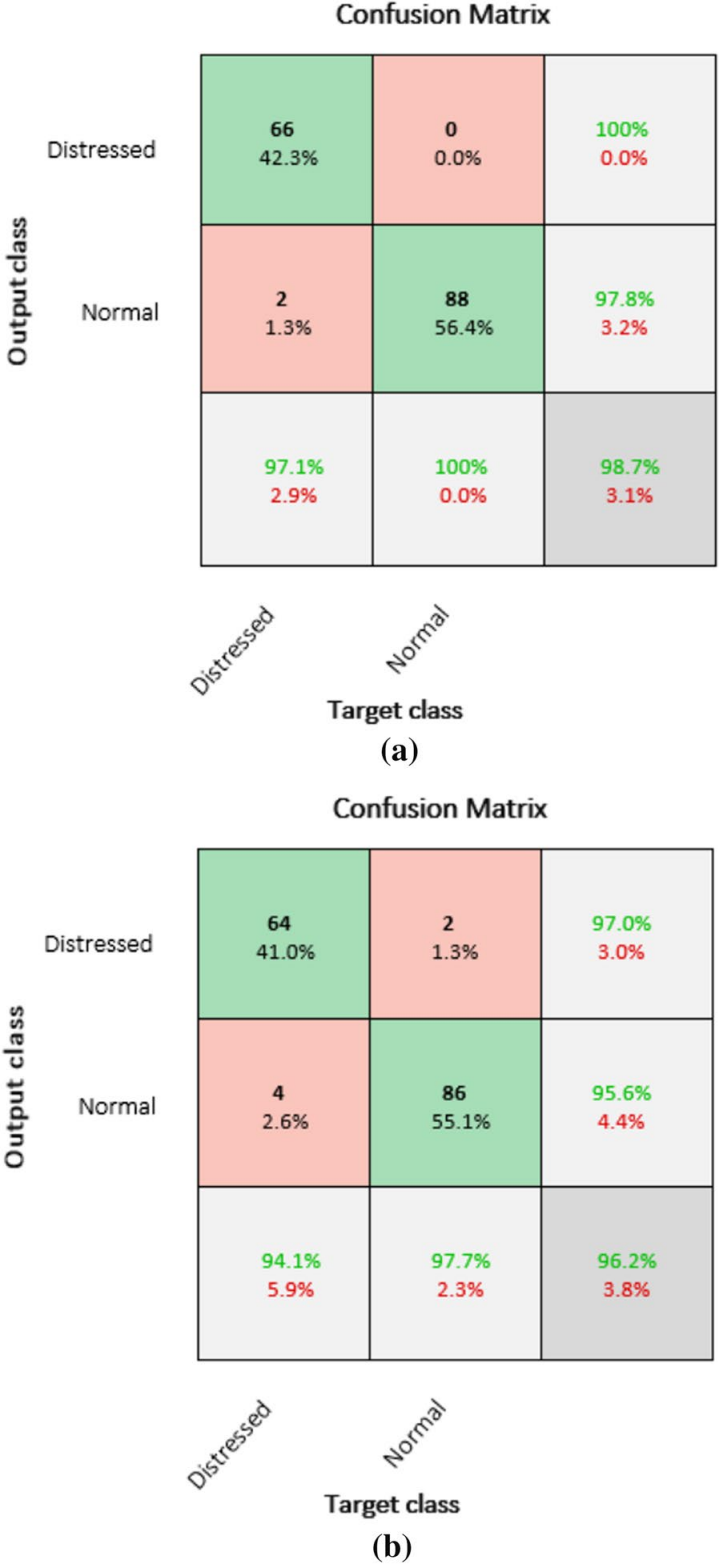
Confusion matrix for experiment 1 (a) and 2 (b)

**Table 2:** Summary of the most related works and current work

Paper	Database Source	Labeling criteria	Stage	Method	Performance
Bursa et al. [16]	CTU-UHB	pH	1st (60 min)	CWT + 2D CNN	Acc: 94.1%
Comert et al. [17]	CTU-UHB	pH	2nd (15 min)	STFT + 2D CNN (AlexNet)	Acc: 93.32%, Sen: 56.15% Sp: 96.51%
Zhao et al. [18]	CTU-UHB	pH	1st (20 min)	CWT + 2D CNN	Acc: 98.34%, Sen: 94.87% Sp: 97.82%
Parvathavarthine et al. [19]	CTU-UHB	pH	2nd (30 min)	Optimized Res Net 50	Acc: 94.63%
Frasch et al. [20]	Private	Visual	N/A	Customized 2D CNN	Acc: 93.6%
Ogasawara et al. [21]	CTU-UHB	Ph&apgar	2nd (30 min)	2D CTG-net	AUC: 0.73
Saini et al. [22]	CTU-UHB	pH	1st (60 min)	FHR + UC + 2D CNN	Acc: 70%, 71.4% and 70%
Current study	CTU-UHB	pH	1st (20 min) and 2nd (15 min)	Morse wavelet + CNN(ResNet50)	Acc: 98.7%, Sen.: 97.0% Sp: 100%
					Acc: 96.1%, Sen.: 94.1% Sp: 97.7%

## Data Availability

The data sets used and/or analyzed during the current study are available from the corresponding author on reasonable request.

## Abbreviations

AI: Artificial intelligence
ANS: Autonomic nervous system
AW: Analytic wavelets
BPM: Beat per minute
CNN: Convolutional neural network
CTG: Cardiotocograms
CAD: Computer aided diagnosis
CWT: Continuous wavelet transform
ECG: Electrocardiogram
EEG: Electroencephalogram
EMG: Electromyogram
FHR: Fetal heart rate
FT: Fourier transform
FECG: Fetal electrocardiogram
FIGO: International Federation of Gynecology and Obstetrics
NIFECG: Non invasive electrocardiogram
RCOG: Royal College of Obstetricians and Gynecologists
ResNet: Residual network
SBR: Still birth rate
STFT: Short time Fourier Transform
UC: Uterine contraction
WHO: World Health Organization
WT: Wavelet transform
